# Inflammasome-Driven Fatal Acute-on-Chronic Liver Failure Triggered by Mild COVID-19

**DOI:** 10.3390/v16101646

**Published:** 2024-10-21

**Authors:** Vivian Chih-Wei Chen, Craig Ryan Joseph, Wharton O. Y. Chan, Wan Rong Sia, Qi Su, Xin Xiu Sam, Hemavathi Tamilarasan, Yun Yan Mah, Wei Lun Ng, Joe Yeong, Lin-Fa Wang, Thinesh L. Krishnamoorthy, Wei-Qiang Leow, Matae Ahn, Wan Cheng Chow

**Affiliations:** 1 Programme in Emerging Infectious Diseases, Duke-NUS Medical School, Singapore 169857, Singaporelinfa.wang@duke-nus.edu.sg (L.-F.W.); 2 Institute of Molecular and Cell Biology (IMCB), Agency for Science, Technology and Research (A*STAR), Singapore 138673, Singapore; 3 Department of Anatomical Pathology, Singapore General Hospital, Singapore 169856, Singapore; 4 Immunology & Serology Section, Department of Microbiology, Division of Pathology, Singapore General Hospital, Singapore 169856, Singapore; 5 SingHealth Duke-NUS Global Health Institute, Singapore 169857, Singapore; 6 Department of Gastroenterology and Hepatology, Singapore General Hospital, Singapore 169608, Singapore; 7 SingHealth Duke-NUS Medicine Academic Clinical Program, Singapore 168753, Singapore; 8 SingHealth Internal Medicine Residency Program, Singapore 169608, Singapore

**Keywords:** inflammasome, NLRP3, ASC, COVID-19, SARS-CoV-2, primary biliary cholangitis, autoimmune hepatitis, acute-on-chronic liver failure

## Abstract

Inflammasome is linked to many inflammatory diseases, including COVID-19 and autoimmune liver diseases. While severe COVID-19 was reported to exacerbate liver failure, we report a fatal acute-on-chronic liver failure (ACLF) in a stable primary biliary cholangitis-autoimmune hepatitis overlap syndrome patient triggered by a mild COVID-19 infection. Postmortem liver biopsy showed sparse SARS-CoV-2-infected macrophages with extensive ASC (apoptosis-associated speck-like protein containing a CARD) speck-positive hepatocytes, correlating with elevated circulating ASC specks and inflammatory cytokines, and depleted blood monocyte subsets, indicating widespread liver inflammasome activation. This first report of a fatal inflammatory cascade in an autoimmune liver disease triggered by a mild remote viral infection hopes to elucidate a less-described pathophysiology of ACLF that could prompt consideration of new diagnostic and therapeutic options.

## 1. Introduction

Inflammasome is a multi-protein complex typically found in monocytes/macrophages. It consists of a sensor (e.g., NLRP3), an adaptor (ASC, apoptosis-associated speck-like protein containing a CARD), and an effector (caspase-1). Upon detecting pathogens or danger signals, the inflammasome induces the release of proinflammatory cytokines (e.g., IL-1β and IL-18) and pyroptosis, an inflammatory form of cell death.

Inflammasome activation has been implicated in the pathogenesis of inflammatory diseases, including viral infections, autoimmune disorders, metabolic conditions, and neurodegenerative diseases [1]. Specifically, NLRP3 inflammasome activation was recently shown to promote chronic cholestatic liver injury and autoimmune hepatitis [2,3]. In COVID-19, inflammasome activation in SARS-CoV-2-infected circulating monocytes and lung tissue-resident macrophages drives inflammation and virus-induced pathology [4,5]. Following SARS-CoV-2 infection with respiratory failure, cirrhotic patients have high rates of hepatic decompensation, acute-on-chronic liver failure (ACLF), and death [6]. However, there has been no evidence that mild self-resolving COVID-19 can precipitate fatal ACLF in stable chronic liver disease.

Here, we report the first case of an unusually rapid clinical deterioration following mild COVID-19 in a stable, cirrhotic patient who had primary biliary cholangitis-autoimmune hepatitis (PBC-AIH) overlap syndrome. Our molecular, cellular, and histological analyses reveal a fatal inflammatory cascade in the liver, likely driven by inflammasome activation with an interplay between SARS-CoV-2 and the underlying autoimmune liver disease.

## 2. Case Report

**Figure 1:**
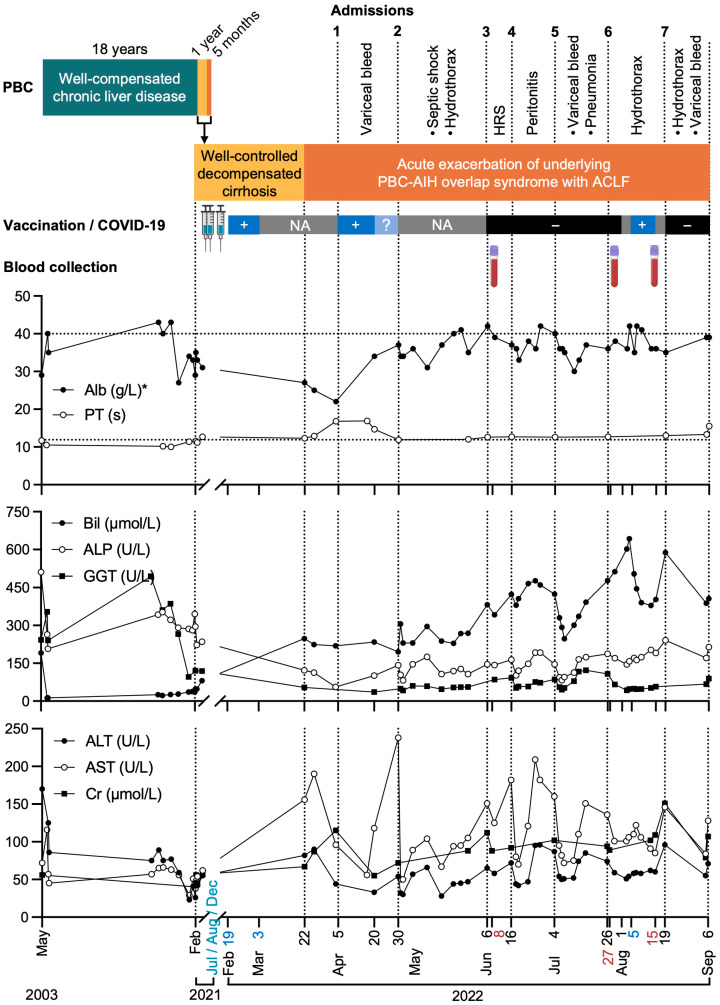
Patient timeline and biochemical tests. Important clinical events, vaccination schedule, and time of blood collection matched with biochemical tests. Syringe, COVID-19 vaccination (cyan); +, COVID-19-positive by ART and/or PCR (dark blue); NA, no available COVID-19 test results (gray); ?, COVID-19-indeterminate by PCR (light blue); −, COVID-19-negative by PCR (black); blood tube, blood collection (red); Alb, albumin; PT, prothrombin time; Bil, bilirubin; ALP, alkaline phosphatase; GGT, gamma-glutamyltransferase; ALT, alanine aminotransferase; AST, aspartate aminotransferase; Cr, creatinine. * Patient received intravenous albumin intermittently as clinically indicated, such as during large-volume abdominal paracentesis or chest drainage for hydrothoraxes. Created with BioRender.com.

A 58-year-old female patient presented with marked ascites on 22 March 2022, following a mild, symptomatically self-limiting COVID-19 upper respiratory tract illness (URTI), self-diagnosed by Antigen Rapid Test (ART) one month prior. She had a background of PBC-AIH overlap syndrome, diagnosed 19 years earlier in 2003. Her condition was managed with ursodeoxycholic acid and azathioprine with partial control of her mildly but persistently deranged liver function tests (LFTs), which was predominantly cholestatic but asymptomatic. She developed ultrasonic features of cirrhosis (Child–Pugh Class B8) and presented with her first episode of mild ascites in February 2021, which was subsequently well controlled with low-dose diuretics. She remained ascites-free on spironolactone 50 mg and furosemide 20 mg daily until post-COVID-19. Meanwhile, she completed her two doses of SARS-CoV-2 mRNA vaccine in July and August 2021 and a booster dose in December 2021 (Figure 1 and Supplementary Figure S1).

In March 2022, she presented to the clinic with a one-week history of recurrent ascites (Figure 1). The patient reported testing ART-positive for 14 days, albeit symptomatically well apart from her initial URTI. She then developed ascites 11 days after becoming ART-negative, despite her usual maintenance diuretic treatment. While she was prescribed an increased dose of diuretics and later intravenous albumin, this was followed by a massive variceal hemorrhage, necessitating hospital admission two weeks later. During this first admission, she also developed diuretic-resistant ascites requiring large-volume abdominal paracentesis (LVAP) and was found to have a new, non-occlusive portal vein thrombosis extending to the proximal superior mesenteric vein. This was followed by six additional admissions over the next five months before her demise.

Aside from an elective admission for pre-liver transplantation investigations, all other hospitalizations were due to complications related to rapidly decompensating liver disease, specifically in the manifestation of portal hypertension, viz. recurrent and rapidly accumulating ascites that required repeated LVAP, spontaneous bacterial peritonitis, right hepatic hydrothorax requiring repeated pleural paracentesis, recurrent variceal hemorrhage that failed endoscopic variceal ligations and required radiology-guided angioembolization, and hepatorenal syndrome associated with hyponatremia. The patient was given intravenous terlipressin and midrodrine. Notably, while there was a marked drop in the patient’s platelet count (trough 32 × 10^9^/mL), the prothrombin time (PT) was relatively normal (11.5–12.7 s) throughout her illness. Apart from a peak of 16.8 s during her first hospital admission for hematemesis, the PT, unsupported by blood products, was 13.3 s the day before her demise.

Although she reported self-tested negative ART results from March 4, 2022, she was found to be SARS-CoV-2 polymerase chain reaction (PCR)-positive during routine testing at her first admission on 5 April 2022 (Figure 1). She later became SARS-CoV-2 PCR-negative spontaneously in subsequent admissions but was SARS-CoV-2 PCR-positive again during routine surveillance testing in her sixth admission, which was not associated with respiratory symptoms. Other than an episode of aspiration pneumonia following her massive variceal hemorrhage, there was no evidence of parenchymal lung disease on serial chest X-rays during her various hospital admissions.

Of note, comparing her pre-COVID-19 baseline LFTs, the most significant difference was the magnitude of progressive increase in serum bilirubin. Conversely, there was no concurrent steady increase in aspartate/alanine transaminases (AST/ALT) or alkaline phosphatase (ALP) (Figure 1). The intermittent isolated increase in AST correlated with the patient’s acute clinical events during her hospital admissions.

Several clinical manifestations of this case were inexplicable from the limited clues of routine clinical tests performed. The mechanisms by which a mild viral infection of COVID-19 could trigger fatal ACLF upon its clinical resolution remains unclear. Additionally, the reasons behind the patient’s markedly accelerated cumulative manifestations of portal hypertension and lone, severe hyperbilirubinemia, despite the lack of other obvious evidence of augmented hepatocellular dysfunction (associated with relatively preserved PT), were unknown. Therefore, serial blood samples during the course of the patient’s illness (Figure 1) and postmortem liver biopsies were taken to answer these questions.

## 3. Materials and Methods

### 3.1. Clinical Specimens

Blood samples were collected in BD Vacutainer^®^ EDTA tubes (BD, Franklin Lakes, NJ, USA). Plasma and peripheral blood mononuclear cells (PBMCs) were separated using SepMate™ (STEMCELL Technologies, Vancouver, BC, Canada) per the manufacturer’s protocol. Postmortem liver biopsies were obtained 1.5 h following the patient’s demise. The biopsies were either fixed in 10% formalin or flash-frozen in isopentane and liquid nitrogen bath before downstream processing.

### 3.2. Histology and Multiplex Immunohistochemistry

Hematoxylin and eosin (H&E) staining (Richard-Allan Scientific LLC, MI, USA) was performed on formalin-fixed paraffin-embedded (FFPE) liver biopsy sections using standard protocols. Multiplex immunohistochemistry (mIHC) was performed on 4 µm thick FFPE tissue sections using Leica Bond Max autostainer (Leica Biosystems, Melbourne, Australia), Bond Refine Detection Kit (Leica Biosystems, Newcastle Upon Tyne, UK), and Opal Fluorophore Reagent Packs (Akoya Biosciences, Marlborough, MA, USA) [7,8,9,10,11]. In brief, FFPE tissue sections were deparaffinized, rehydrated, and subjected to repeated cycles of heat-induced epitope retrieval, incubation with primary antibody (Supplementary Table S1), secondary antibodies conjugated with polymeric HRP (Bond Refine Kit), and Opal tyramide signal amplification (TSA) (Akoya Biosciences). After all markers were applied, spectral DAPI (Akoya Biosciences) was then applied as the nuclear counterstain. Lastly, slides were mounted with ProLong Diamond Antifade Mountant (Molecular Probes, Life Technologies, USA) and cured in the dark at room temperature for 24 h. Images were captured using Zeiss Axioscan 7 slide scanner (Zeiss, Oberkochen, Germany) and analyzed by a pathologist using HALO v3.6 (Indica Labs, Albuquerque, NM, USA) software. CD68^+^ cells and total cells (DAPI^+^) were counted by HALO software, while ASC speck^+^ cells within CD68^+^ or CD68^−^ populations were manually counted in the representative periportal or lobular regions (five regions each).

### 3.3. Bulk RNA Sequencing

RNA from the patient’s fresh-frozen postmortem liver biopsy was extracted through TRIzol™ Reagent (ThermoFisher, Waltham, MA, USA) or RNeasy Mini Kit (QIAGEN, Singapore, Singapore). Both RNA samples were then prepared as libraries with SMART-Seq^®^ v4 Ultra^®^ Low Input RNA Kit (Takara Bio, San Jose, CA, USA) and NEBNext^®^ Ultra™ Direction RNA Library Prep Kit for Illumina (NEB, Ipswich, MA, USA), respectively. Bulk RNA sequencing (RNA-Seq) was performed using NovaSeq6000 (Illumina, San Diego, CA, USA), and reads were aligned to Hg38 using STAR (v2.7.10a) [12] and summarized into count tables using featureCounts in package Rsubread (v2.8.2) [13]. Healthy liver bulk RNA-Seq (*n* = 10, GEO: GSE135251) and PBC liver single-nuclei (sn)RNA-Seq (*n* = 2, GEO: GSE247128) data were downloaded as count tables [14,15]. For snRNA-Seq data, a filter of nUMI > 875, nFeatures > 500, and percentage mitochondrial reads < 50% was applied, as stated in the publication. Pseudo-bulk processing was performed by summation of all cells present across the library. Library size was normalized by calcNormFactors, and targeted differential gene expression analysis was performed using glmQLFTest (edgeR v3.36.0) [16]. Viral reads were extracted using the VirDetect pipeline (https://github.com/dmarron/virdetect (accessed on 2 September 2024)) [17] and plotted as coverage plots using the Gviz package (v1.34.1) [18]. Box plots were created using ggplot2 (v3.3.6) with cpm calculated using the edgeR cpm (x, keep.lib.siz = F) function [19].

### 3.4. SARS-CoV-2 Serology

Neutralizing antibodies against the wild-type SARS-CoV-2 strain were analyzed using cPass™ (GenScript, Piscataway, NJ, USA) per the manufacturer’s protocol.

### 3.5. Cytokine Measurement

Plasma cytokine levels were measured using a 48-plex Luminex cytokine panel (Bio-Rad, Hercules, CA, USA) per the manufacturer’s protocol and analyzed using five-parameter logistic curve fitting.

### 3.6. Flow Cytometry

Recovered PBMCs were blocked with 1% Fc block for 15 min at 4 °C then stained with surface marker antibodies in FACS buffer (phosphate-buffered saline (PBS) supplemented with 2% FBS and 2 mM EDTA) for 30 min at 4 °C in the dark. Cells were fixed for 30 min at 4 °C in the dark before undergoing intracellular staining. Fluorophore-conjugated antibodies used are listed in the Supplementary Table S1. Acquisition was performed using LSRFortessa flow cytometer (BD Biosciences, Franklin Lakes, NJ, USA). Compensation beads or stained cells were used to set up compensation calculations on BD FACSDiva software (v8.0.1). Plasma, initially ranging from 400 to 500 µL (starting volume), was concentrated to 10 µL via centrifugation at 14,000× *g* for 10 min and incubated with 100 µL of PE anti-ASC (TMS-1) antibody for 30 min at room temperature in the dark. After two washes, the stained ASC specks were resuspended in 300 µL, with 100 µL of this volume acquired on CytoFLEX LX cytometer (Beckman Coulter, Brea, CA, USA) using CyExpert software (v2.4). Acquired events were analyzed using FlowJo software (v10) (TreeStar, Ashland, OR, USA). The final concentration of ASC specks in the plasma was calculated using the following equation: final concentration (count/µL) = raw counts × 3/starting volume.

## 4. Results

### 4.1. Liver Response

**Figure 2:**
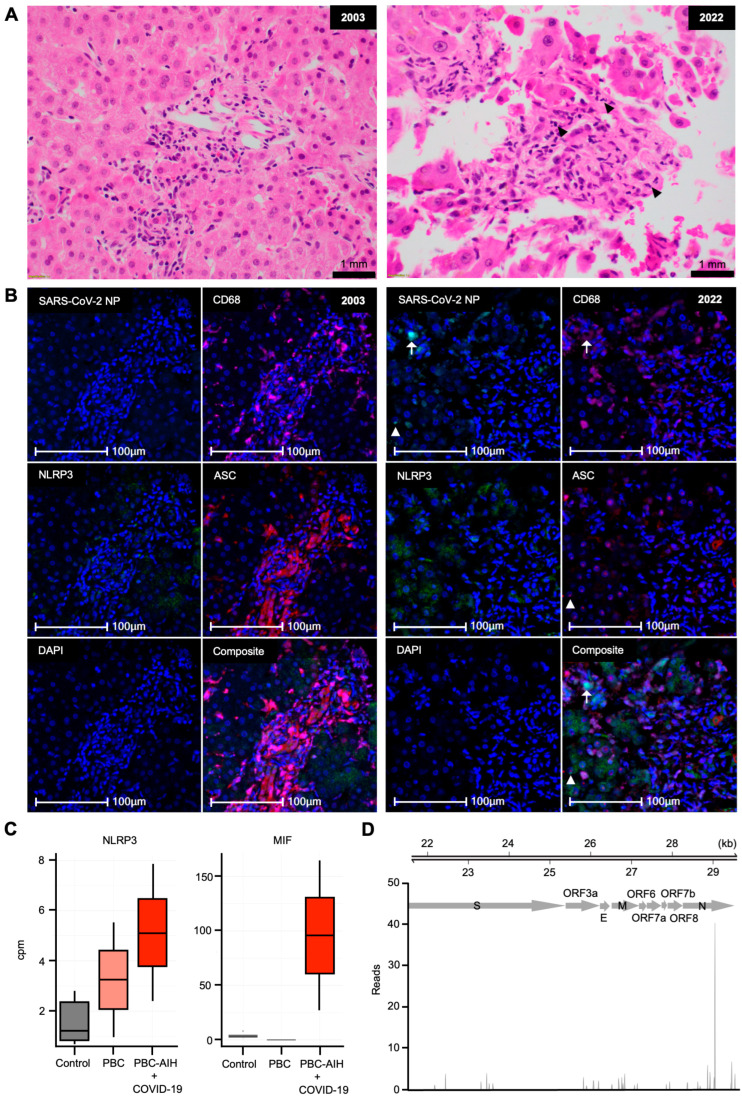
Limited SARS-CoV-2 infection but significant widespread inflammasome activation in the postmortem liver biopsy of the patient. (**A**,**B**) H&E and mIHC of liver biopsy at diagnosis (2003, left) and at time of death (2022, right). Black arrowheads, neutrophils. Cyan, SARS-CoV-2 NP; pink, CD68; green, NLRP3; red, ASC; blue, DAPI; arrows, SARS-CoV-2-infected NP^+^ CD68^+^ cells; arrowheads, NP^+^ ASC speck^+^ cells. Scale bars, 1 mm (**A**) and 100 µm (**B**). (**C**) Counts per million (cpm) of inflammatory genes from bulk RNA-Seq data of patient’s postmortem liver biopsy (red) compared to healthy liver bulk RNA-Seq data (*n* = 10) (gray) and PBC liver snRNA-Seq data (*n* = 2) (pink). (**D**) Mapped reads of SARS-CoV-2 viral genes from bulk RNA-Seq of patient’s postmortem liver biopsy. S, spike; E, envelope; M, membrane; N, nucleocapsid; ORF3a, 6, 7a, 7b, and 8, open reading frame 3a, 6, 7a, 7b, and 8.

The patient’s liver biopsy that was taken at diagnosis in 2003 was retrieved to be reviewed and compared with the postmortem liver biopsy by the same histopathologist. H&E staining of the 2003 biopsy showed mixed inflammatory infiltrates in the portal tracts consisting of lymphocytes, a few plasma cells, and eosinophils. Although granulomas were absent, the overall histological features were consistent with PBC and AIH. H&E staining of the postmortem liver biopsy showed portal tracts with disrupted bile ducts, more cholestasis compared to the 2003 biopsy, and only a slightly more pronounced mixed inflammatory infiltrate with neutrophils, but there were no features to suggest ongoing hepatobiliary sepsis, significant liver injury, or advanced fibrosis (Figure 2A).

Hence, mIHC was performed to specifically examine SARS-CoV-2 infection and inflammasome activation. mIHC of the postmortem liver biopsy revealed a low number of sparsely distributed CD68^+^ macrophages infected by SARS-CoV-2 (~18% of CD68^+^ cells or 2–3% of total cells), as evidenced by positive nucleocapsid (NP) staining (Figure 2B), even though the patient had tested PCR-negative from nasal pharyngeal swabs two weeks prior (Figure 1). CD68^−^ hepatocytes were also found to be infected by SARS-CoV-2 but at a much lower level (~4% of CD68^−^ cells). Enhanced NLRP3 expression in hepatocytes was observed throughout the postmortem biopsy compared to the 2003 biopsy (Figure 2B), indicating a primed inflammatory environment. Importantly, ASC specks, the hallmark of inflammasome activation where diffuse ASC proteins assemble into a single perinuclear protein aggregate, were extensively found in the patient’s postmortem biopsy, around 10–30% of total cells depending on the regions (Figure 2B and Table 1). ASC speck^+^ cells were located near SARS-CoV-2-infected liver macrophages (NP^+^ CD68^+^) (Figure 2B). ASC specks were detected intracellularly in both CD68^+^ macrophages (up to 12% of CD68^+^ cells compared to up to 6% at diagnosis) and CD68^−^ hepatocytes (up to 30% of CD68^−^ cells compared to only up to 0.5% at diagnosis) (Table 1). Co-localization of NPs and ASC specks supports the role of NPs in activating the inflammasome (Figure 2B) [20]. In contrast, ASC was diffusely expressed and more apparent in macrophages than hepatocytes at diagnosis (Figure 2B), with only around 1% ASC speck^+^ cells of total cells (Table 1). Overall, mIHC findings support widespread NLRP3 inflammasome activation in the postmortem liver, possibly triggered by SARS-CoV-2-infected liver macrophages.

To examine the overall liver response, bulk RNA-Seq was performed on the patient’s postmortem liver biopsy. Two key inflammasome-related candidates, NLRP3 and macrophage inhibition factor (MIF), which is required for the NLRP3 inflammasome pathway [21], were elevated in the patient’s liver but not in the PBC alone or healthy liver datasets by targeted differential gene expression analysis (Figure 2C and Supplementary Tables S2 and S3) [14,15]. In addition, most of the other inflammasome-related genes, including ASC, caspase-1, and IL-1β, as well as some inflammatory cytokines such as IL-6, also showed upregulation in the patient’s liver compared to PBC alone liver, again suggesting an inflammatory state (Supplementary Table S3). Furthermore, partial viral genomes/transcripts were detected by read-mapping analysis, but at low coverage and read depth across the SARS-CoV-2 genome (Figure 2D), suggesting a low level of infection correlating with the scarce SARS-CoV-2 NP detected by mIHC (Figure 2B).

### 4.2. Systemic Response

**Figure 3:**
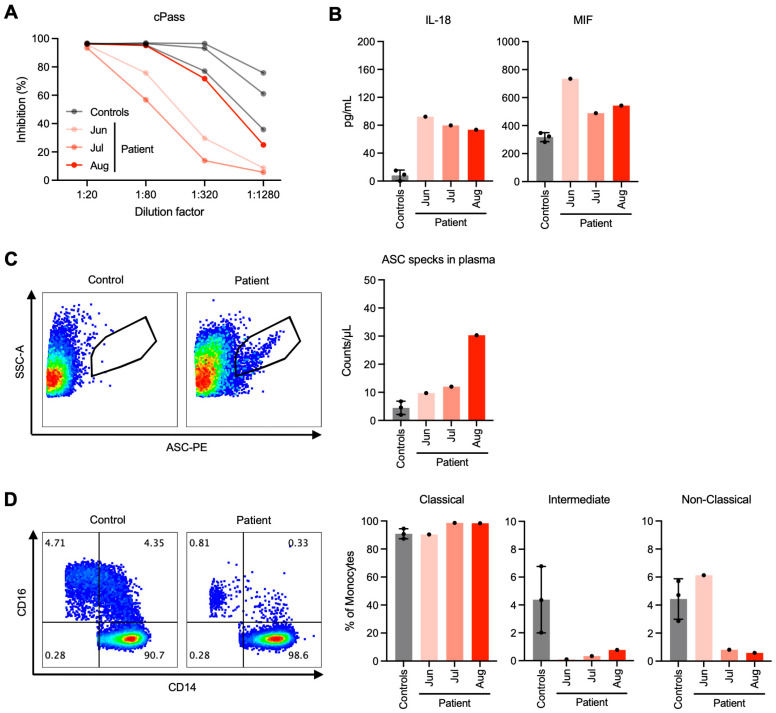
Reduced SARS-CoV-2-neutralizing antibodies, increased proinflammatory cytokines and ASC specks, and depleted monocyte subsets in the peripheral blood of the patient. (**A**,**B**) SARS-CoV-2-neutralizing antibodies by surrogate virus neutralization test (**A**) and proinflammatory cytokines by Luminex (**B**) in the plasma of the patient compared to healthy controls. (**C**,**D**) Gating strategy and quantification of ASC specks (**C**) and monocyte subsets (**D**) by flow cytometry.

To understand the patient’s systemic immune response to COVID-19, a surrogate virus neutralization test [22], cPass™, revealed low neutralizing antibody titers to the wild-type SARS-CoV-2 strain in the patient, despite a more recent full vaccination with booster and multiple SARS-CoV-2 infections compared to healthy controls (Figure 3A and Supplementary Figure S1). This might have predisposed her to recurrent SARS-CoV-2 infections and spreading of the virus to the liver despite clinically mild COVID-19. The relatively improved antibody response in August 2022 was likely a result of the SARS-CoV-2 infection detected on 5 August 2022. The overall poor antibody response of the patient could be attributed to her long-term intake of immunosuppressant drug, albeit at a relatively low dose of a single agent (azathioprine 50 mg/day), and possibly to cirrhosis-associated immune dysfunction [23].

Systemic inflammation assayed by a cytokine panel revealed elevated levels of two proinflammatory cytokines linked to the inflammasome, IL-18 and MIF, in the patient’s plasma compared to healthy controls (Figure 3B). In addition to cytokine release, cells with inflammasome activation can release ASC specks into the extracellular space, thereby propagating inflammation within organs or even to distant organs [24]. By flow cytometry, we detected circulating ASC specks in the patient’s plasma (Figure 3C), possibly originating from extensive inflammasome activation and ASC speck release from the liver. The patient’s peripheral blood cells also showed markedly decreased intermediate (CD14^high^ CD16^+^) and non-classical (CD14^low^ CD16^+^) monocytes (Figure 3D), hinting at elevated cell death or depletion due to their infiltration to the liver in this context of systemic inflammation.

## 5. Discussion

The case presents an unexpected acceleration of clinical deterioration of a PBC-AIH overlap patient following symptomatically mild and clinically resolved COVID-19. Postmortem liver biopsy revealed limited SARS-CoV-2-infected macrophages surrounded by extensive regions of elevated NLRP3 and ASC specks in hepatocytes, indicating a pronounced and widespread inflammasome activation in the liver. This observation was consistent with circulating ASC specks, inflammatory cytokines, and decreased monocyte subsets detected in the blood. Cirrhotic patients with COVID-19-induced respiratory failure are known to be more susceptible to hepatic decompensation/failure. While infection was the most common predisposing factor of ACLF, they tend to be bacterial in nature [25]. There was a report of influenza virus causing ACLF in cirrhotic patients, but the mechanism of which was not alluded [26]. Sterile inflammation that precipitates ACLF was observed in the background of exacerbation of patients’ underlying liver disease [27]. Therefore, the mechanisms by which resolving, mild COVID-19 leading to hepatic decompensation in the background of stable PBC-AIH overlap syndrome remain unclear yet intriguing.

The patient’s recurrent COVID-19 was likely due to a low titer of SARS-CoV-2-neutralizing antibodies, as demonstrated by cPass™. While PCR tests of nasopharyngeal swabs were negative two weeks prior to the patient’s demise, SARS-CoV-2 remnants or a very low level of infection likely persisted in the liver, supported by the presence of a low amount of NP staining on mIHC and the detection of a low level of partial viral RNA sequences. This might have been predisposed by her long-term immunosuppressant usage and cirrhosis-associated immune dysfunction. There is similar evidence of persistence of SARS-CoV-2 RNA in body fluids [28,29]. The SARS-CoV-2 antigens detected in the patient’s postmortem liver biopsy were found in some of the macrophages, which likely originated from infiltrating monocytes infected directly by SARS-CoV-2 [30] and/or via uptake of the immune complex of SARS-CoV-2 complexed with antibodies [4,5]. While monocytes/macrophages can be infected by SARS-CoV-2, the nature of the infection is abortive with no live virus progeny [4,5], which is the likely reason for the patient’s limited hepatocyte infection considering hepatocytes’ propensity for infection [31]. Although the patient’s SARS-CoV-2 infection was not directly spread to hepatocytes, the infected and activated macrophages could still lead to priming and activation of the inflammasome in the surrounding hepatocytes, as evident by the increased NLRP3 expression and ASC specks on mIHC. Resident or infiltrated macrophages, once activated either directly by viral infection [30], or indirectly via the immune complex of SARS-CoV-2 [4,5,32], could secrete pro-inflammatory cytokines and release ASC specks after pyroptosis, which in turn would prime and activate the hepatocytes. The overall decrease in macrophage numbers in the postmortem liver might be contributed by cell death due to increased inflammasome activation in macrophages and/or loss of liver macrophages in the process of cirrhosis. The relatively intact state of hepatocytes could be due to their resistance to pyroptosis [33], explaining the patient’s relatively unaffected liver synthetic function during the clinical deterioration. This is in contrast to other common causes of ACLF secondary to sepsis where bacteria can cause other forms of cell death or severe COVID-19 with viremia leading to direct hepatocyte infection and cell death [31].

Interestingly, while the rest of the patient’s LFTs (including ALP) and synthetic function (suggested by PT) appeared relatively preserved, the patient’s lone and severe hyperbilirubinemia might suggest impaired bilirubin transport and/or conjugation in these activated hepatocytes. In addition, an increased level of bile acid could contribute to a vicious cycle of cholestasis aggravated by NLRP3 inflammasome activation. It was probably not a coincidence that the inflammasome activation triggered by mild COVID-19 resulted in such a devastating outcome in this patient, considering the patient’s background of chronic cholestasis and potentially low-grade NLRP3 activity in association with PBC [2]. In this case, the elevated bile acid probably acted as a damage-associated molecular pattern and/or a second signal in perpetuating the inflammasome activation [34,35]. Despite the lack of pyroptosis, a low amount of proinflammatory cytokine produced by hepatocytes could prime the surrounding hepatocytes to induce a homogenous upregulation of NLRP3 [33], recruit and activate more inflammatory cells, and induce a wider spread of inflammation. The widespread inflammasome activation in hepatocytes and immune cell infiltration might lead to an increased vascular tone in the liver, which could be an explanation for the worsening of the patient’s portal hypertension on top of the original inflammatory and prothrombotic state induced by COVID-19.

Inflammasomes have been implicated in autoimmune liver diseases, including chronic cholestatic diseases and AIH [2,3]. In such cases, testing for anti-viral antibody titers may help guide prophylactic vaccination schedules in patients with chronic liver diseases, especially the cirrhotics. Additionally, educating patients on health-seeking behaviors can ensure early initiation of antivirals despite mild symptoms of non-hepatic conditions. Developing better diagnostics, such as ASC specks and other inflammasome biomarkers, may be useful in monitoring seemingly covert inflammatory activity and disease severity to guide prompt clinical management decisions. New therapeutics targeting inflammasomes (e.g., ASC specks) might be crucial in changing the trajectory of disease progression for patients at risk of ACLF [36].

Limitations are to be noted in this study. In terms of the patient’s liver disease progression, we were only able to compare the liver biopsy at the time of death to the time of initial diagnosis. The patient’s blood analyses were directly compared to healthy controls instead of PBC-AIH patients without COVID-19, making it difficult to determine the extent of inflammation caused by COVID-19 and PBC-AIH individually. Nonetheless, to our knowledge, this is the first documented case of accelerated liver failure post-mild SARS-CoV-2 infection.

## Figures and Tables

**Table 1:** Quantification of ASC speck^+^ and/or CD68^+^ cells in the patient’s postmortem liver biopsy (2022) compared to initial diagnosis (2003).

Year	2003		2022	
Region	Lobular	Periportal	Lobular	Periportal
CD68^+^ of total cells (%)	13.2	19.7	8.7	15.0
ASC speck^+^ of total cells (%)	1.0	0.9	28.7	12.1
ASC speck^+^ of CD68^+^ cells (%)	6.0	2.2	12.2	3.6
ASC speck^+^ of CD68^−^ cells (%)	0.2	0.5	30.3	13.6
CD68^+^ of ASC speck^+^ cells (%)	83.3	52.2	3.7	4.4
CD68^−^ of ASC speck^+^ cells (%)	16.7	47.8	96.3	95.6

## Data Availability

The raw data supporting the conclusions of this article will be made available by the authors on request.

## Supplementary Materials

The following supporting information can be downloaded at https://www.mdpi.com/article/10.3390/v16101646/s1, Table S1: Key resources; Table S2: List of DEGs from comparison between healthy control and PBC-AIH with COVID-19 liver RNA sequencing datasets; Table S3: List of DEGs from comparison between PBC and PBC-AIH with COVID-19 liver RNA sequencing datasets; Figure S1: COVID-19 vaccination and infection history of the PBC-AIH patient and healthy controls.
