# Supplementary material for: Inflammasome-Driven Fatal Acute-on-Chronic Liver Failure Triggered by Mild COVID-19
Source: Viruses. 2024 Oct 21;16(10):1646. doi: 10.3390/v16101646 (PMC11512379; doi:10.3390/v16101646)
Supplement: Supplementary file 1 [file viruses-16-01646-s001.zip › PBC Supp_Proof_Final.pdf]

SUPPLEMENTARY MATERIALS

Inflammasome-Driven Fatal Acute-on-Chronic Liver Failure Triggered by Mild COVID-19

Vivian Chih-Wei Chen [1,†], Craig Ryan Joseph [2,†], Wharton O. Y. Chan [1,†], Wan Rong Sia [1], Qi Su [1], Xin Xiu Sam [3], Hemavathi Tamilarasan [2], Yun Yan Mah [1], Wei Lun Ng [1], Joe Yeong [2,4], Lin-Fa Wang [1,5], Thinesh L. Krishnamoorthy [6], Wei-Qiang Leow [3], Matae Ahn [1,7,8,*], Wan Cheng Chow [6,7,*]

Affiliations

[1] Programme in Emerging Infectious Diseases, Duke-NUS Medical School, 169857 Singapore

[2] Institute of Molecular and Cell Biology (IMCB), Agency for Science, Technology and Research (A*STAR), 138673 Singapore

[3] Department of Anatomical Pathology, Singapore General Hospital, 169856 Singapore

[4] Immunology & Serology Section, Department of Microbiology, Division of Pathology, Singapore General Hospital, 169856 Singapore

[5] SingHealth Duke-NUS Global Health Institute, 169857 Singapore

[6] Department of Gastroenterology and Hepatology, Singapore General Hospital, 169608 Singapore

[7] SingHealth Duke-NUS Medicine Academic Clinical Program, 168753 Singapore

[8] SingHealth Internal Medicine Residency Program, 169608 Singapore

† These authors contributed equally to this work.

* Correspondence: ahn.matae11@u.duke.nus.edu; Tel.: +65 8482 0217 (M.A.); chow.wan.cheng@singhealth.com.sg; Tel.: +65 8125 3601 (W.C.C.)

Formatted: Position: Horizontal: 11.22 cm, Relative to: Page, Vertical: 0 cm, Relative to: Paragraph

Table S1. Key resources

REAGENT or RESOURCE	SOURCE	IDENTIFIER
**Antibodies**		
Anti-Human SARS-Nucleocapsid protein, rabbit polyclonal	Novus Biologicals	Cat# NB100-56576; RRID: AB_838838
Anti-Human CD68, mouse monoclonal (PG-M1)	Dako Agilent	Cat# M0876; RRID: AB_2074844
Anti-NLRP3/NALP3, mouse monoclonal Ab (Cryo-2)	AdipoGen	Cat# AG-20B-0014-C100; RRID: AB 2490202
Anti-ASC/TMS1, rabbit monoclonal (E1E3I)	Cell Signaling Technology	Cat# 13833; RRID: AB_2798325
CD14-FITC	invitrogen	Cat# 11-0149-42; RRID: AB_10597597
CD19-PE-Cy5	BioLegend	Cat# 302210; RRID: AB_314240
Va7.2-PE-Cy7	BioLegend	Cat# 351712; RRID: AB_2561994
CD161-APC-Cy7	BioLegend	Cat# 3399228; RRID: AB_2563967
HLA-DR-BUV395	BD Horizon™	Cat# 564040; RRID: AB_2738558
CD8-BV570	BioLegend	Cat# 301038; RRID: AB_2563213
CD3-BV650	BioLegend	Cat# 300468; RRID: AB_2629574
CD16-AF700	BD Pharmingen™	Cat# 560713; RRID: AB_1727430
CD56-BUV737	BD Horizon™	Cat# 564447; RRID: AB_2744432
CD4-BV510	BioLegend	Cat# 357419; RRID: AB_2715939
ASC-PE	BioLegend	Cat# 653903; RRID: AB_2564507
Granzyme B-BV421	BD Horizon™	Cat# 563389; RRID: AB_2738175
Active Caspase-3-AF647	BD Pharmingen™	Cat# 560626; RRID: AB_1727414
LIVE/DEAD™ Fixable Blue Dead Cell Stain Kit	Invitrogen™	Cat# L23105
eBioscience™ Foxp3 / Transcription Factor Staining Buffer Set	Invitrogen™	Cat# 00-5523-00
Human TruStain FcX™ (Fc Receptor Blocking Solution)	BioLegend	Cat# 422302; RRID: AB_2818986
**Chemicals**		
Antibody Diluent, Background Reducing	Dako Agilent	Cat# S302283
TRIzol™ Reagent	ThermoFisher	Cat# 15596026
**Commercial assays**		
Bond Refine Detection Kit	Leica Biosystems, Newcastle Upon Tyne, UK	Cat# DS9800
Opal Fluorophore Reagent Packs	Akoya Biosciences, Marlborough, MA, USA	https://www.akoyabio.com/phenoimager/assays/opal-fluorophore-reagent-packs/
RNeasy Mini Kit	QIAGEN	Cat# 74104
NEBNext® Ultra™ Direction RNA Library Prep Kit	NEB	Cat# NEB #E7760
SMART-Seq® v4 Ultra® Low Input RNA Kit	Takara Bio	Cat# 634894

Formatted: Position: Horizontal: 11.22 cm, Relative to: Page, Vertical: 0 cm, Relative to: Paragraph

SepMate™ PBMC Isolation Tubes	STEMCELL	Cat# 86415
cPass™ SARS-CoV-2 Neutralization Antibody Detection Kit	GenScript	https://www.genscript.com/covid-19-detection-fda-eua.html
Bio-Plex Pro Human Cytokine Screening Panel, 48-Plex	Bio-Rad	Cat# 12007283
**Deposited data**		
Bulk RNA-Seq: Healthy liver	Govaere et al.	GEO: GSE135251
snRNA-Seq: PBC liver	Andrews et al.	GEO: GSE247128
**Software and algorithms**		
GraphPad Prism (v9)	GraphPad Software	RRID: SCR_002798; http://www.graphpad.com/
HALO 3.6	Indica Labs	RRID:SCR_018350; https://www.indicalab.com/halo/
R Project for Statistical Computing (v4.1.3)	The R Foundation	RRID: SCR_001905; http://www.r-project.org/
STAR (v 2.7.10a)	Dobin et al.	RRID: SCR_004463; http://code.google.com/p/rna-star/
Rsubread (v 2.8.2)	Liao et al.	RRID: SCR_016945; https://bioconductor.org/packages/release/bioc/html/Rsubread.html
edgeR (v 3.36.0)	Robinson et al.	RRID:SCR_012802; http://bioconductor.org/packages/edgeR/
Gviz package (v 1.34.1)	Hahne et al.	RRID:SCR_024239; https://bioconductor.org/packages/Gviz/
ggplot2 (v 3.3.6)	Wickham	RRID: SCR_014601; https://cran.r-project.org/web/packages/ggplot2/index.html
BD FACSDiva™ Software (v8.0.1)	BD BioSciences	RRID: SCR_001456; http://www.bdbiosciences.com/instruments/software/facsdiva/index.jsp
CytExpert Software (v2.4)	Beckman Coulter	RRID:SCR_017217; https://www.beckman.fr/flow-cytometry/instruments/cytoflex/software
FlowJo (v10)	TreeStar	RRID: SCR 008520; https://www.flowjo.com/

Table S2. List of DEGs from comparison between healthy control and PBC-AIH with COVID-19 liver RNA sequencing datasets.

See file: PBC COVID_to_Control_DEGs.xlsx.

Table S3. List of DEGs from comparison between PBC and PBC-AIH with COVID-19 liver RNA sequencing datasets.

See file: PBC COVID_to_PBC_DEGs.xlsx.

Formatted: Position: Horizontal: 11.22 cm, Relative to: Page, Vertical: 0 cm, Relative to: Paragraph

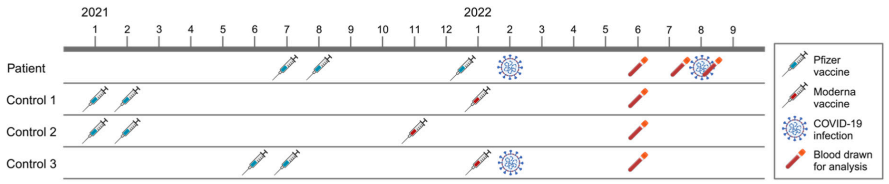

Figure S1. COVID-19 vaccination and infection history of the PBC-AIH patient and healthy controls. Created with BioRender.com.

Formatted: Position: Horizontal: 11.22 cm, Relative to: Page, Vertical: 0 cm, Relative to: Paragraph
